# Molar pregnancy in the perimenopausal period: a case report

**DOI:** 10.11604/pamj.2025.52.61.49131

**Published:** 2025-10-05

**Authors:** Aloui Haithem, Eya Azouz, Hatem Frikha, Rami Hammami, Bilel Arfaoui, Feriel Slama, Selma Dhaoui, Saber Hassine Abouda, Badis Chanoufi

**Affiliations:** 1 Faculté de Médecine de Tunis Service C, Centre de Maternité et de Néonatologie de Tunis, Université Tunis El Manar, Tunis, Tunisie

**Keywords:** Molar pregnancy, perimenopause, gestational trophoblastic disease, hemorrhagic shock, case report

## Abstract

Molar pregnancy is an uncommon diagnosis in perimenopausal women and poses a high risk of complications, including hemorrhagic shock and malignant transformation. We report the case of a 51-year-old woman who presented with severe abdominal pain and profuse vaginal bleeding. Imaging revealed a markedly enlarged uterus with a large intracavitary mass, and serum β-hCG levels were elevated. The patient developed hemorrhagic shock and underwent an emergency hemostatic hysterectomy. Histopathological examination confirmed a complete hydatidiform mole with early invasive features, without choriocarcinoma. Postoperative imaging showed no metastases, and the patient received methotrexate-based chemotherapy with favorable response, evidenced by a progressive decline in β-hCG levels. This case underscores the importance of considering gestational trophoblastic disease in the differential diagnosis of abnormal uterine bleeding in perimenopausal women. Early recognition and timely surgical intervention, followed by appropriate chemotherapy and monitoring, are crucial to avoid potentially life-threatening complications.

## Introduction

Gestational trophoblastic disease (GTD) encompasses a spectrum of disorders ranging from benign hydatidiform mole to malignant entities such as invasive mole and choriocarcinoma [1]. Although GTD is most common in women of reproductive age, rare cases occur in perimenopausal women, in whom the diagnosis may be delayed due to overlapping symptoms with menopausal transition [2,3]. Molar pregnancy in this age group carries an increased risk of persistent trophoblastic neoplasia and complications such as severe hemorrhage [4,5]. We report a rare case of complete molar pregnancy with early invasive features in a 51-year-old woman, complicated by hemorrhagic shock and managed with emergency hysterectomy followed by chemotherapy.

## Patient and observation

**Patient information:** a 51-year-old woman, gravida 9 para 4 (four term vaginal deliveries), with a medical history of Graves´ disease, presented to the emergency department in August 2025 with abdomino-pelvic pain and heavy vaginal bleeding ongoing since April 2025. Her last menstrual period was on February 22, 2025.

**Clinical findings:** on physical examination, the patient appeared pale and in distress. Her blood pressure was 85/50 mmHg, pulse rate 118 beats/min, respiratory rate 24 cycles/min, and temperature 37.2°C. Oxygen saturation was 92% on room air. The conjunctivae were markedly pale, consistent with acute anemia. Abdominal examination revealed a distended uterus with tenderness, while pelvic examination showed a gravid-appearing cervix and profuse vesicular metrorrhagia.

**Timeline of current episode:** the patient began experiencing abnormal uterine bleeding in April 2025, which progressively worsened over several months. Her last menstrual period was on February 22, 2025. In early August 2025, she presented to the emergency department with acute abdomino-pelvic pain and heavy vaginal bleeding. A pelvic ultrasound performed on admission revealed an enlarged uterus with a large, heterogeneous intracavitary mass. A pre-admission magnetic resonance imaging (MRI), conducted at a private facility, confirmed a markedly enlarged uterus with a voluminous hemorrhagic and microcystic mass. Shortly after admission, the patient developed hemorrhagic shock and was taken for emergency hemostatic hysterectomy. Histopathological analysis confirmed a complete hydatidiform mole with early invasive changes. A postoperative computerized tomography (CT) scan performed within the first week excluded metastatic disease. She was started on methotrexate chemotherapy (1 mg/kg on days 1, 3, 5, and 8), receiving three cycles. By August 10, 2025, her serum β-hCG level had decreased to 55.8 mIU/mL, and she remained under outpatient follow-up.

**Figure 1:**
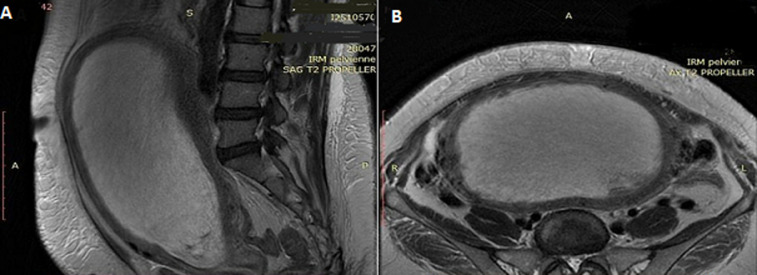
sagittal and axial magnetic resonance imaging sections showing an enlarged uterus with a microcystic and hemorrhagic intracavitary process

**Diagnostic assessment:** transabdominal pelvic ultrasound revealed an enlarged uterus with a 20×11 cm intracavitary heterogeneous echogenic mass, without pelvic fluid. Serum qualitative β-hCG was positive. An MRI performed prior to admission showed a markedly enlarged uterus measuring 26×11.5×18 cm, with a large intracavitary mass (24×8.7×16.6 cm) obliterating the endometrial cavity. The endometrium was not visualized, and the junctional zone was displaced but continuous (Figure 1).

**Diagnosis:** histopathological analysis of the surgical specimen revealed a complete hydatidiform mole with early invasive features and no evidence of choriocarcinoma. Postoperative thoraco-abdomino-pelvic CT scan showed no signs of metastatic disease.

**Figure 2:**
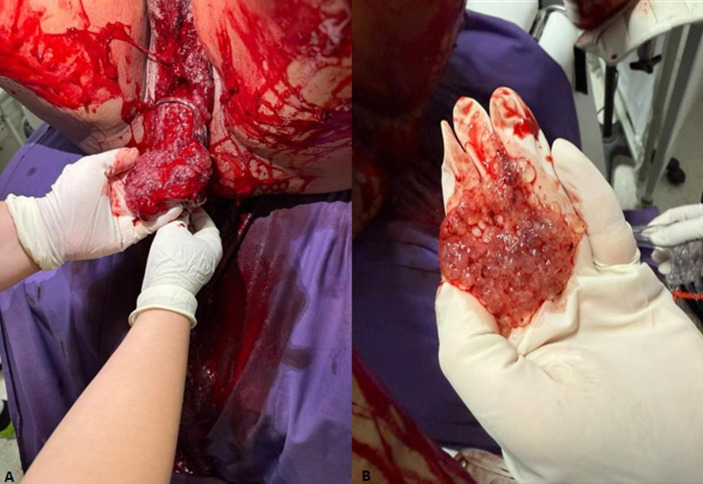
massive vesicular metrorrhagia

**Therapeutic interventions:** shortly after hospital admission, the patient developed hemorrhagic shock and underwent an emergency hemostatic hysterectomy (Figure 2). Postoperatively, she was started on methotrexate-based chemotherapy (1 mg/kg administered on days 1, 3, 5, and 8), completing three cycles.

**Follow-up and outcomes:** serial monitoring of serum β-hCG was performed postoperatively to assess treatment response. On postoperative day 7, the level was 12,500 mIU/mL, decreasing to 2,800 mIU/mL after the first cycle of methotrexate. Following the second cycle, β-hCG further declined to 340 mIU/mL, and after the third cycle it reached 55.8 mIU/mL. This progressive reduction reflected a favorable therapeutic response. Clinically, the patient remained stable, was discharged on postoperative day 3, and is currently being followed up in an outpatient setting with ongoing β-hCG surveillance to ensure complete remission.

**Patient perspective:** the patient expressed surprise upon learning that she had a molar pregnancy at her age. She described her experience as emotionally and physically challenging, especially due to the sudden need for emergency surgery. However, she reported feeling well-informed and supported by the care team throughout the process, and expressed relief at the favorable outcome and close follow-up.

**Informed consent:** written informed consent was obtained from the patient for the publication of this case report and the accompanying images.

## Discussion

Molar pregnancy, a subtype of gestational trophoblastic disease (GTD), is characterized by abnormal trophoblastic proliferation and is classified into complete and partial hydatidiform moles based on genetic and histopathological features. Complete hydatidiform moles, as observed in our case, result from fertilization of an anucleate ovum by one or two sperm, leading to a diploid paternal genome without maternal contribution [1]. Although the overall incidence of complete mole is estimated at 1-3 per 1,000 pregnancies in developed countries [2], it is exceedingly rare in women over 50 due to the natural decline in fertility. Most reported cases occur in the 40-50 age group, and postmenopausal presentations are exceptional, often associated with high risks of invasive mole or choriocarcinoma [3,4]. Advanced maternal age is a well-established risk factor, likely due to oocyte anomalies and chromosomal instability in older women [2,3]. Additional risk factors include previous molar pregnancies and extremes of maternal age [2,5]. Typical clinical signs include abnormal uterine bleeding, uterine enlargement disproportionate to gestational age, and elevated β-hCG levels [1,6]. However, in perimenopausal women, such symptoms can be misattributed to other conditions such as menopausal transition, endometrial hyperplasia, or uterine malignancy, delaying diagnosis [4,7].

In our case, the presence of Graves' disease introduced an additional diagnostic challenge, given that β-hCG can stimulate thyroid receptors and rarely lead to thyrotoxicosis in complete moles [1]. Diagnostic imaging was critical: pelvic ultrasound identified the heterogeneous echogenic mass, and MRI further confirmed a large intracavitary lesion obliterating the endometrial cavity, but with a preserved junctional zone, an important finding in assessing myometrial invasion [6]. A striking feature of this case was the abrupt progression to hemorrhagic shock, precipitated by massive vesicular bleeding and uterine distension. Although rare, hemorrhagic shock in molar pregnancy is documented in the literature and is linked to the friable vascular nature of molar tissue or coagulopathy [8]. In older women, these risks are amplified by reduced physiological reserve and comorbidities [3]. Emergency hysterectomy was justified by the patient´s age, completed parity, and hemodynamic instability. This approach is consistent with guidelines favoring hysterectomy in perimenopausal women with complete moles, as it reduces tumor burden and lowers malignant transformation risk, which can exceed 50% in this age group [3,9,10]. Pathology confirmed early invasive features without choriocarcinoma, and imaging ruled out metastases, supporting the appropriateness of the intervention.

Despite hysterectomy, 15-20% of patients develop persistent gestational trophoblastic neoplasia (GTN) [1,9]. In our case, persistently elevated β-hCG prompted initiation of methotrexate, the first-line treatment for low-risk GTN. This regimen is associated with 70-90% response rates and is generally well tolerated [7]. The patient responded favorably, with β-hCG declining steadily after chemotherapy. In case of methotrexate resistance, actinomycin-D may be used as second-line therapy [7,9]. Close monitoring is essential to detect relapse, which occurs in 3-5% of cases, especially among patients with invasive disease or advanced age [2,9]. Current guidelines recommend weekly β-hCG monitoring until normalization (<5 mIU/mL for three consecutive weeks), followed by monthly testing for 6-12 months depending on disease severity [9,10]. Our patient is currently under such surveillance. This case illustrates the diagnostic and therapeutic complexities of molar pregnancy in perimenopausal women and underscores the need for high clinical vigilance, timely imaging, and a multidisciplinary approach to avoid life-threatening complications.

## Conclusion

This case underscores the critical need to include gestational trophoblastic disease in the differential diagnosis of abnormal uterine bleeding in perimenopausal women, despite its rarity in this age group. Early recognition, guided by appropriate imaging and β-hCG testing, is essential to avoid severe complications such as hemorrhagic shock. In high-risk patients with completed childbearing, emergency hysterectomy remains a life-saving intervention and may reduce the risk of malignant transformation. When indicated, adjuvant chemotherapy with methotrexate contributes to excellent disease control. Multidisciplinary management and close post-treatment monitoring are key to achieving favorable outcomes in this vulnerable population.
